# Mechanism of ferroptosis regulating ischemic stroke and pharmacologically inhibiting ferroptosis in treatment of ischemic stroke

**DOI:** 10.1111/cns.14865

**Published:** 2024-07-23

**Authors:** Zhaohui Chai, Jiesheng Zheng, Jian Shen

**Affiliations:** ^1^ Department of Neurosurgery First Affiliated Hospital, College of Medicine, Zhejiang University Hangzhou City China

**Keywords:** ferroptosis, iron, ischemic stroke, lipid peroxide, ROS

## Abstract

Ferroptosis is a newly discovered form of programmed cell death that is non‐caspase‐dependent and is characterized by the production of lethal levels of iron‐dependent lipid reactive oxygen species (ROS). In recent years, ferroptosis has attracted great interest in the field of cerebral infarction because it differs morphologically, physiologically, and genetically from other forms of cell death such as necrosis, apoptosis, autophagy, and pyroptosis. In addition, ROS is considered to be an important prognostic factor for ischemic stroke, making it a promising target for stroke treatment. This paper summarizes the induction and defense mechanisms associated with ferroptosis, and explores potential treatment strategies for ischemic stroke in order to lay the groundwork for the development of new neuroprotective drugs.

## Graphical abstract

Ferroptosis plays a pivotal role in the pathogenesis of cerebral infarction, where the delicate balance between pro‐ and anti‐ferroptotic factors critically influences the outcomes of an ischemic stroke. Our review aims to comprehensively elucidate the underlying mechanisms of ferroptosis and explore therapeutic strategies targeting ferroptosis in ischemic stroke.

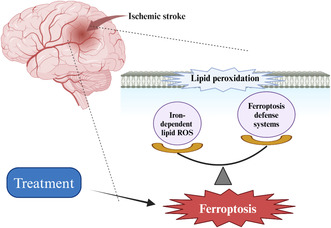

## 1 INTRODUCTION

The term ferroptosis was coined in 2012 by Dixon et al.^1^ It is characterized by the generation of lethal levels of iron‐dependent lipid ROS and is associated with oxidative stress and inflammation. Lipid peroxides are considered to be the primary inducers of ferroptosis, while other compounds that stimulate the production of cytoplasmic or mitochondrial ROS do not trigger ferroptosis.^2^ Ferroptosis is different from other forms of programmed cell death such as necrosis, apoptosis, autophagy, and pyroptosis in morphology, physiology, and genetics. For instance, from a morphological perspective, ferroptotic cells lack characteristic features such as chromatin aggregation and margination, organelle swelling, and plasma membrane rupture. The primary morphological hallmarks include shrunken mitochondria, increased membrane density, and decreased mitochondrial cristae numbers.^1^, ^3^ Ferroptosis occurs when the balance between pro‐ferroptotic and anti‐ferroptotic factors is disrupted.^4^ Ferroptosis can be induced by the accumulation of glutamate, iron, or polyunsaturated fatty acid (PUFA) − phospholipids, or the consumption of endogenous ferroptosis inhibitors such as GSH, NADPH, glutathione peroxidase 4 (GPX4), or vitamin E.^5^

Ferroptosis, as a unique form of cell death, has aroused great attention in the medical community, and has been associated with various diseases, including neurological diseases (such as ischemic stroke,^6^, ^7^ cerebral hemorrhage,^8^ subarachnoid hemorrhage,^9^ amyotrophic lateral sclerosis,^10^ Parkinson's disease,^11^ etc.), cancer,^12^, ^13^ cardiovascular diseases,^14^ and metabolic diseases.^15^ Globally, stroke is the third leading cause of death and the primary cause of disability worldwide, with ischemic stroke accounting for more than 70% of strokes.^16^ Currently, treatment goals for ischemic stroke focus on salvaging the penumbra, such as thrombolysis, mechanical thrombolysis, etc.; however, these approaches have limited efficacy due to their time window constraints. There is an urgent need for more research into stroke. This review paper summarizes recent advancements in understanding potential regulatory mechanisms of ferroptosis. Additionally, we discuss the potential pathophysiological role of ferroptosis in ischemic stroke and possible treatment strategies.

## 2 THE DISCOVERY OF FERROPTOSIS

Stockwell and his colleagues conducted a synthetic lethal screening of key RAS‐related targets through high‐throughput sequencing. Approximately 24,000 compounds were screened, leading to the identification of erastin, which was found to induce non‐apoptotic cell death in Ras‐expressing cells.^17^ They further discovered that erastin induces non‐apoptotic cell death in tumor cells with activating mutations in the RAS–RAF–MEK pathway by binding to voltage‐dependent anion channels 2 and 3 (VDAC2/3), although VDAC alone is not sufficient for erastin function.^18^ Subsequently, Stockwell expanded the screening to approximately 48,000 compounds and identified two small molecules, named RSL3 and RSL5, which increased mortality in the presence of cancer‐causing RAS. While RDL5 acts through VDAC, RSL3 functions independently of VDAC due to its association with elevated iron levels observed in many cancer patients.^19^ The authors studied erastin, RSL3, and RSL5 together. Antioxidants and iron chelators were found to inhibit cell death caused by these three lethal compounds, independent of caspase, and all three compounds induced iron‐dependent, RAS–RAF–MEK‐dependent, oxidative, non‐apoptotic cell death.^20^

The selenoprotein family of glutathione peroxidases (GPx's) converts lipid peroxides and hydrogen into their corresponding alcohols,^21^ Seiler et al.^22^ found that specific knockout of GPX4, a member of the selenoprotein family of glutathione peroxidases, triggers a unique cell death pathway associated with lipid peroxides. Eagle^23^ proposed in 1955 that cysteine is necessary for cellular growth. The Xc‐system is a cystine/glutamate exchange transporter, a heterodimer composed of Solute carrier family 7 member 11 (SLC7A11, also known as xCT) and its chaperone CD98/4F2hc (SLC3A2),^24^ xCT expression on the cell membrane is essential for intracellular GSH synthesis through cystine uptake, and Banjac et al.^25^ demonstrated that the overexpression of xCT provides cellular protection against lipid peroxidation‐induced cell death. Erastin disrupts the uptake of cystine by cells through binding to the Xc‐ system, leading to the accumulation of lipid ROS. This accumulation of ROS, which is heavily reliant on iron, results in a form of cell death distinct from apoptosis, necrosis and autophagy, termed ferroptosis by Stockwell et al.^1^

## 3 THE MOLECULAR MECHANISM OF FERROPTOSIS

**FIGURE 1:**
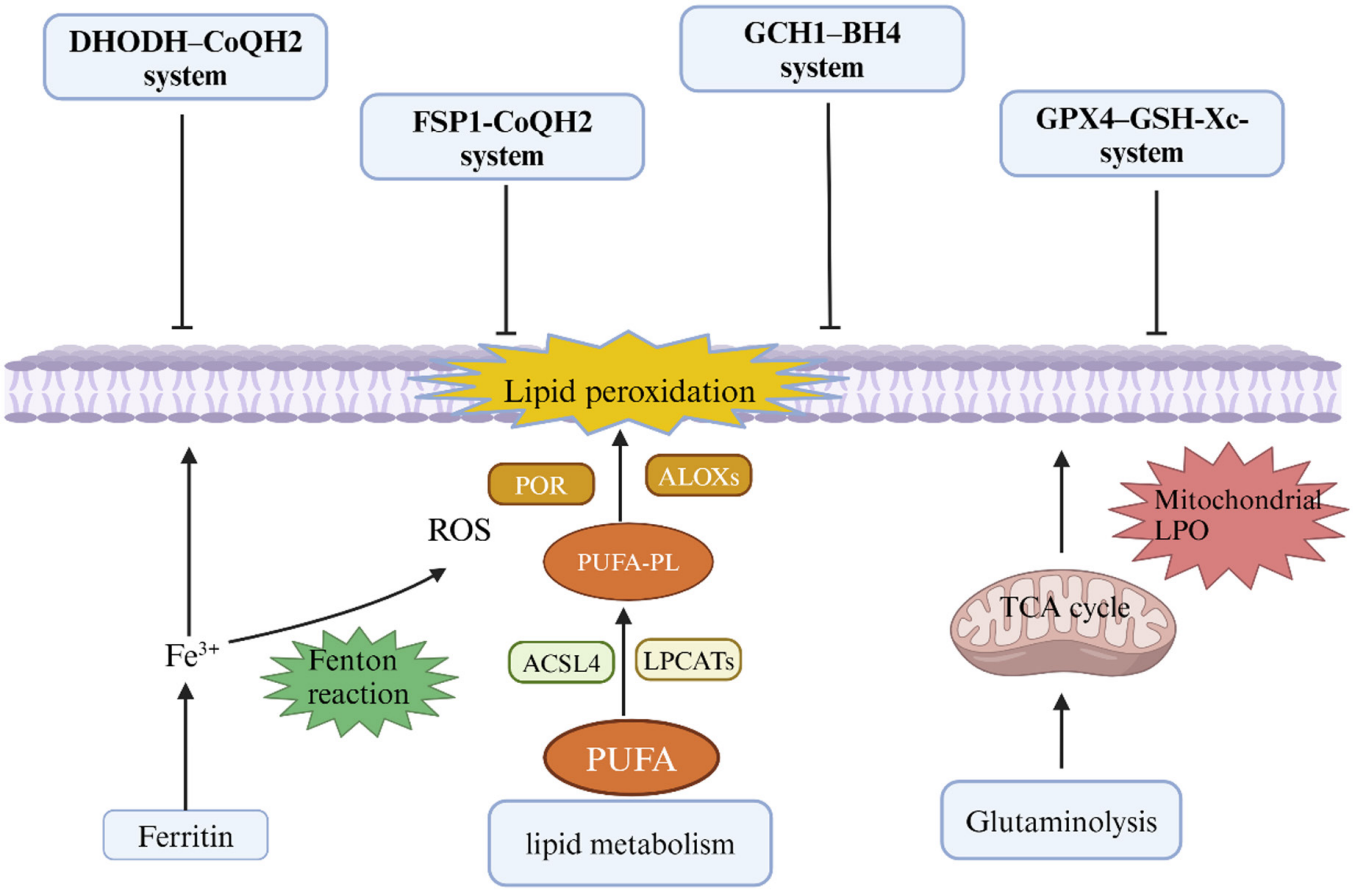
Effects of metabolic processes on cellular susceptibility to ferroptosis. Inadequate regulation of PUFA‐enriched phospholipids and intracellular iron storage is essential for the initiation of ferroptosis, resulting in cell death. The activation of the TCA cycle in mitochondria through glutaminolysis may significantly contribute to the induction of ferroptosis. Ferroptosis is governed by a minimum of four distinct defense mechanisms that shield cells from undergoing ferroptotic cell death.

Ferroptosis is related to three primary hallmarks: iron metabolism, lipid peroxidation and mitochondrial metabolism. The dysregulation of the balance between ferroptosis defense systems and inducers of ferroptosis leads to the initiation of ferroptotic cell death (Figure 1).

### 3.1 Iron homeostasis

Iron is an essential nutrient primarily absorbed by duodenal cells and essential for various biological processes, including enzymatic reactions, mitochondrial function, and DNA synthesis. The interconversion between Fe^2+^ and Fe^3+^ contributes to its effectiveness in biological systems.^26^ However, unbound iron can lead to oxidative damage at subcellular, cellular, and organ levels if it does not bind to specific iron storage proteins or serum carriers such as transferrin or ferritin.^27^ Studies by Stockwell et al.^1^ have shown that erastin or RSL3 disrupt the antioxidant system by promoting intracellular iron accumulation, leading to increased oxidative damage by producing harmful levels of hydroxyl radicals through Fenton reaction.^28^ The oxidation capacity of Fe^2+^ is five times greater than that of Fe^3+^, and the consentration of hydroxyl radicals is proportional to the concentration of Fe^2+^.^29^ Fe^2+^ is oxidized to Fe^3+^ by ceruloplasmin or hephaestin in plasma and binds to transferrin (Tf) in the blood.^30^ Ceruloplasmin (Cp), a glycoprotein with six tightly bound copper ions, belongs to the multicopper oxidase family.^31^ The ferroxidase activity of Cp was initially reported in the early 1960s by Curzon.^32^ Ceruloplasmin inhibits the production of reactive oxygen species mediated by Fe^2+^ through Fenton reaction, serving as a protective agent against oxidative stress.^33^ Additionally, Cp facilitates the excretion of Fe^2+^ from cells via ferroportin exporter and plays a crucial role in iron homeostasis.^34^ Transferrin, a glycoprotein capable of reversibly binding iron in the blood for transport to various tissues and organs, maintains normal physiological activities within these structures. TF can bind not only iron ions (Fe^3+^) but also other metal ions such as copper, manganese, and zinc with different affinities. The iron‐binding properties of TF are pH‐dependent; at neutral pH, iron tightly binds to extracellular TF, while it is released from TF under acidic conditions.^35^ Almost all cellular iron uptake is dependent on the TF/transferrin receptor 1 (TFR1) complex, which undergoes endocytosis after binding to TFR1.^36^ Within endosomes characterized by an acidic environment, Fe^3+^ dissociates from TF before being immediately reduced to Fe^2+^ by ferric reductases six transmembrane epithelial antigen of prostate 3 (STEAP3) or duodenal cytochrome b (DCYTB).^37^ Subsequently transported across endosomal membranes into cytosol via bivalent metal transporter 1 (DMT1),^38^ free Fe^2+^ forms a labile iron pool (LIP) upon binding with GSH within the cytoplasm. Iron chaperones poly (rC)‐binding protein 1/2 (PCBP1/2) are indispensable multifunctional proteins that regulate the storage, utilization, and efflux of iron.^39^, ^40^ Iron levels in the brain are primarily controlled by the blood–brain barrier (BBB).^41^ Iron enters brain microvascular endothelial cells in two forms: holo‐Tf and non‐transferrin binding iron. The binding of the holo‐T complex to TfR and its endocytosis mediated by the reticular protein are the main pathways of iron transport across the luminal membrane.^42^ Iron can form non‐transferrin‐bound complex with citrate and other compounds, which cross the blood–brain barrier faster than transferrin‐bound iron,^43^ however, their physiological relevance remains unclear.^28^ Similarly, Fe^3+^ can also bind to ferritin, there are two subtypes of ferritin, heavy chain FtH and light chain FtL. FtH possesses iron oxide enzyme activity that oxidizes ferrous (Fe^2+^) to Fe^3+^ to facilitate the rapid absorption of iron ions, while FtL is involved in assembling the ferritin core and mainly functions in long‐term storage. Iron is stored as Fe^3+^ within the ferritin molecule, effectively preventing it from participating in the oxidation react.^44^ Ferritinophagy degrades the ferritin, increases the labile iron pool in cells and drives ferroptosis.^45^

Upon entry into the cell, a significant portion of iron is transported into the mitochondria, traversing both the outer membrane (OMM) and inner membrane (IMM) to reach the mitochondrial matrix for heme and iron–sulfur (Fe–S) cluster synthesis. The translocation of iron across the outer mitochondrial membrane is believed to be mediated by DMT1 (also known as SLC11A2),^46^, ^47^ while its passage through the inner mitochondrial membrane is thought to be associated with mitochondrial iron transporter‐1 (MFRN1, also known as SLC25A37) and mitochondrial iron transporter‐2 (MFRN2, also known as SLC25A28).^48^, ^49^ However, there may exist alternative mechanisms for iron transport into mitochondria.^50^ Additionally, iron is absorbed into the bloodstream from intestinal cells, macrophages, and hepatocytes via the iron transporter FPN (also known as SLC40A1).^51^

Iron homeostasis is maintained by a complex network of regulation systems. The absorption, transportation and storage of iron at the translational level are mediated by iron regulatory protein (IRP) 1 or cytoplasmic aconitase (encoded by ACO1) and the iron‐responsive element binding protein 2 or IRP2 (Regulated by the IREB2) pathway.^52^ IRPs are RNA‐binding proteins that bind to cis‐regulated iron response elements (IREs), which are stem‐loop RNA structures located at the 3′ or 5′ UTR of target transcripts, to regulate ferritin, FPN translation, and TfR mRNA stability. IRP1/2 binds to the conserved IREs of FTH, FTL, and FPN mRNAs, and acts as both a translation enhancer and \inhibitor to regulate their translation.^53^ Additionally, IRP interacts with multiple IREs on TFRC mRNAs to protect the mRNAs from degradation.^54^

As previously mentioned, ferritin‐bound iron can be released through the process of ferritinophagy. In conditions of iron deficiency, the multifunctional autophagy receptor nuclear coactivator receptor 4 (NCOA4) recognizes ferritin and binds to the autophagic protein MAP1LC3/LC3 (microtubule associated protein 1 light chain 3). The phagosomes merge with lysosomes to degrade ferritin, release labile iron, and maintain the intracellular labile iron pool.^55^ Therefore, ferritinophagy is largely influenced by the amount of cellular NCOA4, which fluctuate with changes in iron level. When iron is sufficient, HERC2 ubiquitin ligase recruits the ferritinophagy receptor NCOA4 for ubiquitination and subsequent proteasomal degradation, thereby regulating ferritin autophagy at a basal level.^56^ There are also alternative mechanisms for modulating NCOA4 levels. For example, analysis of the NCOA4 promoter reveals typical hypoxia response elements through which hypoxia inducible factor 2a (HIF2a) can regulate NCOA4 levels during ischemic hypoxia and iron deficiency.

Oxygen free radicals generated by Fenton reaction pose a significant threat to cellular and organismal health. NRF2 serves as an important transcription factor for maintaining metabolic, protein and REDOX homeostasis.^57^ Normally, NRF2 is bound to Kelch‐like epichlorohydrin‐associated protein 1 (Keap1), which interacts with actin and undergoes degradation via ubiquitination. However, under condition of oxidative stress or other toxic insults, oxidation or alkylation of cysteine thiol in KEAP1^58^ prevents the degradation of NRF2 by ubiquitination, leading to its translocation into the nucleus where it binds to antioxidant responsive elements (AREs). These AREs are present in the promoters of numerous genes encoding antioxidant and detoxification enzymes that regulate xCT. Nrf2 influences the translation of ARE‐related genes about glutathione synthesis and the expression of HO‐1, and Gpx4 and NADPH quinone oxidoreductase 1 (NQO1) related to REDOX regulation.^59^, ^60^ Furthermore, NRF2 plays a role not only in antioxidation but also in iron storage, exportation, heme synthesis, and hemoglobin catabolism. As a transcription factor, Nrf2 governs the transcription of transferrin receptor (TFR1), FTH1 and Ftl related to iron storage while reducing labile iron levels,^61^ simultaneously regulating the expression of iron transporter protein (FPN1) related to iron exportation.^62^ ATP‐binding cassette subfamily B member 6 (ABCB6) is involved in heme anabolism by facilitating the transport of porphyrins to mitochondria. In hepatogenic cell lines, NRF2 binds to the promoter regions of ABCB6 and regulates its transcription. Ferrochelatase (FECH) catalyzes the insertion of ferrous iron into protoporphyrin IX to generate heme cofactor; similarly, NRF2 modulates the transcriptional activity of FECH.^63^ Senescent erythrocytes undergo phagocytosis and degradation by macrophages, leading to the catalysis of heme into free iron (Fe^2+^), carbon monoxide (CO), and biliverdin by heme oxygenase (HO‐1). The released ferrous iron is recycled while biliverdin undergoes further metabolism. NRF2 functions as a transcription factor to regulate the expression of HO‐1.^64^

As mentioned above, HIF can modulate the level of NCOA4, thereby influencing ferritinophagy. Under normal conditions, the α chains of HIF1 and HIF2 undergo hydroxylation by proline hydroxylase domain enzyme (PHD) and subsequent degradation through the ubiquitin‐proteasome pathway.^65^, ^66^ However, under hypoxic and iron‐deficient conditions where PHD function is limited, stable α‐β dimers of HIF are formed to regulate iron metabolism, erythropoiesis, angiogenesis, glycolysis.^66^, ^67^, ^68^ HIF1 and HIF2 act as transcriptional regulators for TF (encoding transferrin), DMT1 (also known as SLC11A2), FPN (also known as SLC40A1), TFRC, HO‐1, and thus affect iron metabolism.^69^, ^70^, ^71^, ^72^, ^73^

The hepcidin–ferroportin axis: Hepatic, a hepatic hormone, is a small peptide produced in the liver and secreted into the bloodstream. It plays a crucial role in controlling the iron homeostasis.^74^ FPN (ferroportin, also known as SLC40A1) is the sole known mammalian iron exporter and hepcidin induces its degradation of ferroportin by ubiquitination.^75^ FPN is primarily expressed on the reticuloendothelial system where macrophages recycle iron from senescent erythrocytes' hemoglobin degradation.^74^ Hepcidin restricts iron entry from intestinal enterocytes, macrophages and other cells into the plasma while facilitating its removal from the plasma membrane by binding to the ferroportin, the sole iron‐export protein.^73^, ^76^, ^77^ The hepcidin–FPN regulatory axis controls systemic iron homeostasis and ensures sufficient cellular iron supply. Hepcidin expression decreases in several diseases associated with iron overload such as inefficient erythropoiesis (e.g., thalassemia) or hereditary hemochromatosis (HH), whereas excessive hepcidin can lead to anemia of inflammation or iron‐refractory iron deficiency anemia (IRIDA).^78^ RNF217 is an E3 ubiquitin ligase that induces polyubiquitination and degradation of FPN, meanwhile Tet1 (a member of the 10‐11 translocation methylcytosine dioxygenase family of enzymes) mediates the demethylation of the Rnf217 promoter leading to the up‐regulation of RNF217 expression.^79^

### 3.2 Lipid peroxidation

Phospholipid peroxides (PLOOH) represent a distinct category of ROS, its' accumulation can result in the disruption of plasma membrane integrity, leading to ferroptosis.^80^ Iron‐dependent lipid peroxidation involves free radical reactions, wherein hydrogen atoms are extracted from phospholipids containing polyunsaturated fatty acids (PUFA‐PL) to produce phospholipid peroxide (PLOOH). This process is conducted by enzymatic or non‐enzymatic lipid peroxidation reactions.^81^ Enzymes involved in the incorporation of PUFAs into phospholipids (PLs) play an important role in ferroptosis. ACSL4 (a member of the Acyl‐CoA synthetase long‐chain family) serves as a critical drivers of ferroptosis. PUFAs, particularly arachidonic acid (C20:4) and adrenic acid (C22:4), are conjugated with CoA by ACSL4 to produce acyl‐CoA, which is then re‐esterified in phospholipids to generate PL through lysophosphatidylcholine acyltransferase (LPCATs).^82^, ^83^ The mammalian arachidonate lipoxygenases (ALOXs), comprising 6 members containing non‐heme iron (ALOXE3, ALOX5, ALOX12, ALOX12B, ALOX15, and ALOX15B), catalyze the oxidation of PUFAs.^84^ For example, ALOX5 can convert arachidonic acid (AA) into 5‐hydroperoxyeicosatetraenoicacid (5‐HPETE), a precursor of leukotrienes.^80^ In addition, Cytochrome P450 reductase (POR)‐mediated lipid peroxidation serves as a surrogate mediator of ferroptosis. POR is a NADPH‐dependent redox enzyme containing flavin mononucleotide, previously implicated in the maintenance of cellular redox homeostasis and the regulation of lipid desaturase^85^ and elongase^86^ activities. POR facilitates electron transfer from NADPH to microsomal cytochrome P450.^87^, ^88^ Additionally, POR, NADPH oxidase (NOX), NADH‐cytochrome b5 reductase (CYB5R1), mitochondrial electron transport chain pathways and iron‐dependent Fenton reaction collectively generate H_2_O_2_, which subsequently yields free hydroxyl radicals in the presence of ferrous iron and disrupts membrane integrity by peroxidation of PUFA‐PL.^89^ LPO or its derivatives such as 4‐HNE, MDA perforate the plasma membrane or organelle membrane, ultimately instigating cell death.^90^, ^91^, ^92^

### 3.3 Mitochondrial metabolism

**FIGURE 2:**
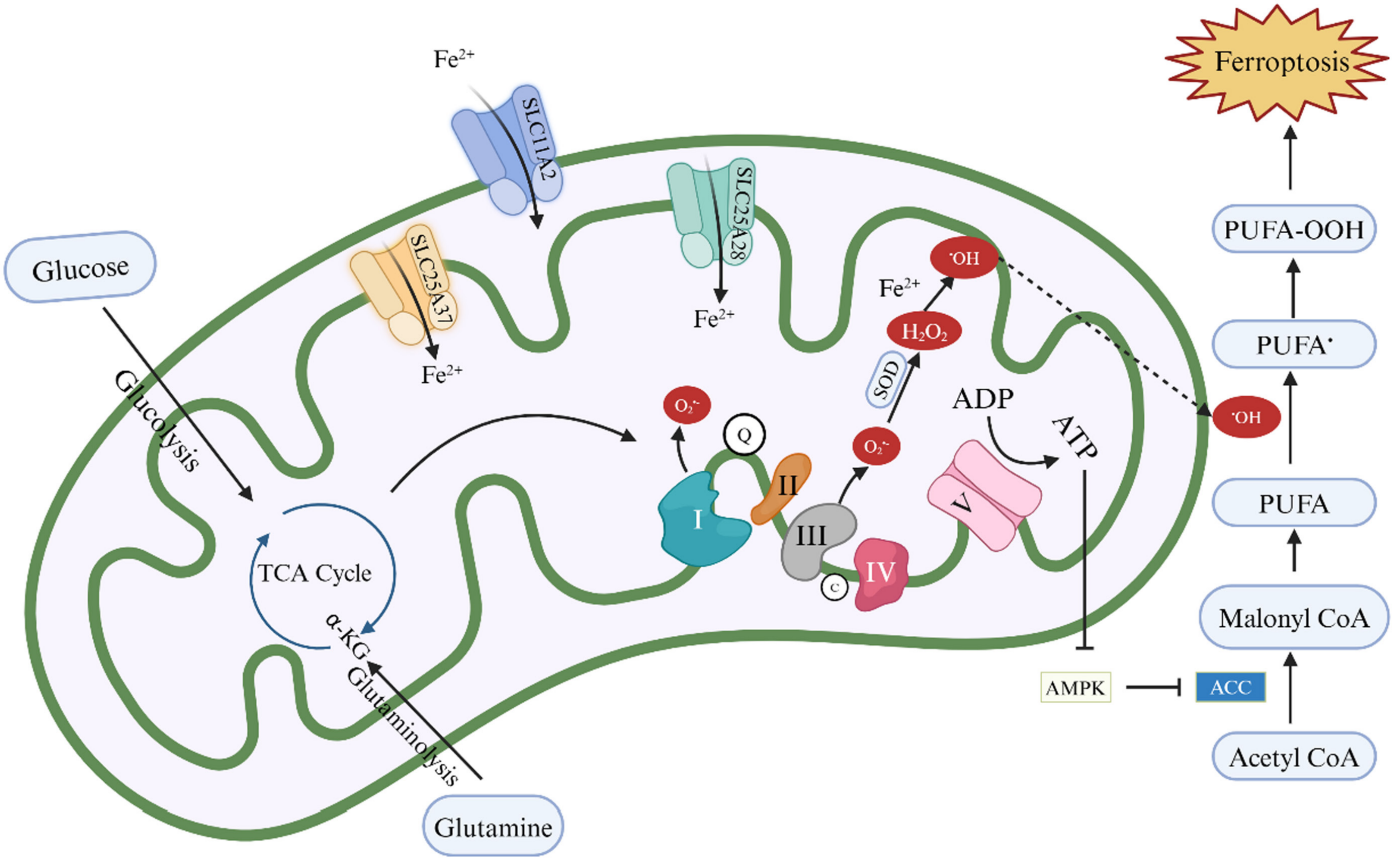
The role of mitochondria in ferroptosis. Fe^2+^ is transported into the mitochondria by SLC11A2, SLC25A37, and SLC25A28. Glutaminolysis and the TCA cycle in the mitochondria are associated with ETC activity and further promote ferroptosis. AMPK inhibits ACC‐mediated conversion of acetyl‐CoA to malonyl‐CoA, thereby ameliorating ferroptosis. Mitochondrial ETCs synthesize ATP to deactivate AMPK, thus promoting ferroptosis. Additionally, mitochondrial ETC complexes I and III generate O2^•‐^, which can enhance PUFA^•^ production and induce ferroptosis.

Several metabolic processes in mitochondria play crucial roles in triggering ferroptosis.^93^ Mitochondria contribute to ferroptosis through involvement in biosynthesis, bioenergetics and ROS regulation. First, mitochondrial ROS production may contribute to ferroptosis by promoting lipid peroxidation as they are the primary source of ROS in cells.. Specifically, electron leakage from ETC complexes I and III produces superoxide, which is subsequently converted to H_2_O_2_ by superoxide dismutase (SOD)‐mediated dismutase. H_2_O_2_ reacts with labile ferrous ion (Fe^2+^) via the Fenton reaction to form hydroxyl radicals (•OH), leading to extraction of diallyl hydrogen in PUFA and formation of PUFA radical (PUFA•). These unstable carbon‐centered radicals rapidly react with oxygen to form PUFA hydroperoxides (PUFA‐OOH).^93^ In addition, electron transport and proton pumping in mitochondria play a significant role in inducing ferroptosis.^94^ Futhermore, mitochondria are pivotal organelles for ATP production. In short, electrons transported through the ETC complex generate proton power that is then utilized by ATP synthase for energy generation.^95^ In the context of energy depletion, activation of the energy sensor AMP‐activated protein kinase (AMPK) leads to phosphorylation and inactivation of acetyl‐CoA carboxylase (ACC), ultimately inhibits the synthesis of some PUFAs and ferroptosis. Conversely, under conditions of abundant energy, AMPK is deactivated, thereby facilitating ferroptosis.^96^ Furthermore, mitochondrial involvement in biosynthesis can also lead to ferroptosis. The tricarboxylic acid (TCA) cycle is the primary source of cellular energy and is involved in a variety of metabolic pathways. Maintenance of TCA cycle function relies on mitochondrial metabolism due to their close interrelation.^97^ Supplementation with glutamine or various anaplerotic reactions that fuel the tricarboxylic acid cycle, such as glutaminolysis, can enhance ferroptosis.^98^ Ketoglutaric acid (αKG), a downstream metabolite of glutaminolysis within the TCA cycle, has been shown to induce lipid ROS accumulation and promote ferroptosis by substituting for glutamine. Additionally, the TCA metabolites downstream of the αKG, including fumarate, succinate and malate, are capable of substituting for glutamine in ferroptosis.^94^, ^99^ (Figure 2).

### 3.4 Ferroptosis defense systems

There are at least four such ferroptosis defense systems with unique subcellular localization as described below.

#### 3.4.1 The GPX4–GSH system

Gpx4 is a member of the GPX protein family responsible for converting cytotoxic lipid peroxides to non‐toxic lipid alcohols by transforming glutathione (GSH) to oxidized glutathione (GSSG),^100^ thereby preserving cell membrane integrity.^101^ Glutathione reductase (GSR) can convert GSSH back to GSH by FAD (flavin adenine dinucleotide) prosthetic group and NADPH, thus contributing to the maintenance of glutathione (GSH) homeostasis and cellular protection against oxidative damage.^102^, ^103^ In various in vitro and in vivo conditions, genetic ablation or pharmacological inhibition of Gpx4 can lead to uncontrolled lipid peroxidation and trigger severe ferroptosis.^4^, ^104^ The levels of GPX4 protein are regulated by the transcription factor Nrf2 or SP2. Supplementation with intracellular glutathione or selenium upregulates GPX4 activity, while post‐translational modifications (PTMs) such as ubiquitination, phosphorylation, succinization, and glycosylation affect the protein level and activity of GPX4.^105^ It was previously believed that only cytoplasmic GPX4 played a role in defending against ferroptosis.^106^ However, recent studies have shown that mitochondrial GPX4 also plays a crucial role in defending against ferroptosis.^107^

The Xc^−^ system comprise xCT and SLC3A2, with xCT serving as the transporter subunit.^108^ xCT exhibits antiporter activity by importing extracellular cysteine into the intracellular space while exporting intracellular glutamate.^109^ Through its promotion of glutathione biosynthesis, Xc^−^ system can protect cells from ferroptosis.^110^

#### 3.4.2 The FSP1‐CoQH2 system

Previous studies have indicated that ferroptosis is regulated only by GPX4^4^ and free radical trapping antioxidants.^111^, ^112^ However, recent studies have shown that the FSP1‐CoQ10‐NAD (P)H pathway exists as a separate parallel system that operates in parallel with GPX4 and glutathione to inhibit phospholipid peroxidation and ferroptosis.^113^ Ferroptosis suppressor protein 1 (FSP1, also known as AIFM2), located at the plasma membrane, is a transcriptional target for NRF2.^113^, ^114^ FSP1 acts as an NAD(P) H‐dependent oxidoreductase to convert Coenzyme Q_10_ (also known as ubiquinone) to CoQ_10_‐H2 (ubiquinol),^115^ The redox activity of CoQ enables it to participate in the mitochondrial electron transport chain by transferring electrons from oxidative phosphorylated (OxPhos) complexes I and II to complex III.^115^ In addition to its role in electron transport, CoQ can trap lipid peroxyl radicals, thereby inhibiting lipid peroxidation and ferroptosis.^113^ While most CoQ is synthesized in the inner mitochondrial membrane, some may be produced outside the mitochondria.^115^

#### 3.4.3 The DHODH–CoQH2 system

The DHODH‐CoQH2 system is a mitochondrial defense mechanism that operates in parallel with the GPX4 system to prevent lipid peroxidation and ferroptosis when GPX4 is inhibited.^116^ DHODH, located in the mitochondria, serves as a rate‐limiting enzyme for pyrimidine nucleotide synthesis, linking this process to the electron transport chain (ETC) at complex III via the CoQ (ubiquinone) pool.^117^ Ubiquinol (CoQ10‐H2), an anti‐ferroptotic lipophilic antioxidant, is generated during the catalytic conversion of dihydroorotate to orotate by DHODH,^117^ akin to the function of FSP‐1 mediated conversion of ubiquinone (CoQ_10_) to CoQ_10_‐H2 in the cytoplasm.^113^

#### 3.4.4 The GCH1–BH4 system

**FIGURE 3:**
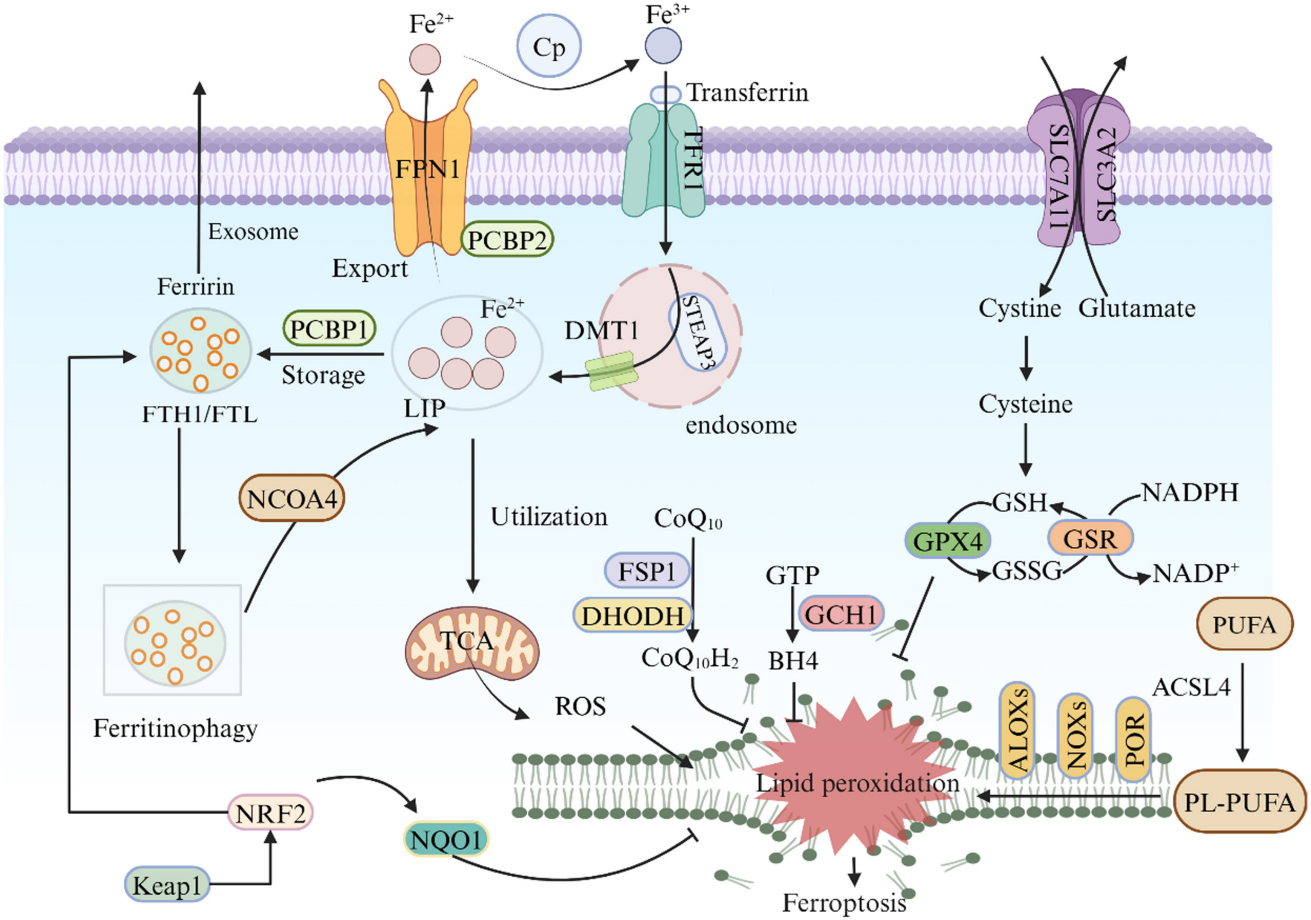
Mechanisms of ferroptosis. Ferroptosis is primarily induced by iron‐dependent lipid peroxidation. All facets of iron metabolism, encompassing absorption, export, storage and utilization, play a pivotal role in the regulation of ferroptosis. The cystine/glutamate transporter, also known as system Xc^−^, facilitates the transport of cystine into the cell with 1:1 glutamate reverse transport. Once inside the cell, cystine can be oxidized to cysteine. The GSH‐GPX4 antioxidant system plays a crucial role in safeguarding cells against ferroptosis. ACSL4 and LPCAT3 promote the binding of PUFAs to phospholipids, forming phospholipids containing PL‐PUFA, which are susceptible to free radical‐induced oxidation mediated by lipoxygenases (ALOXs). In addition, coenzyme Q10 or tetrahydrobiopterin (BH4) inhibits ferroptosis independently of glutathione.

The guanosine 5′‐triphosphate (GTP) cyclohydrolase 1 (GCH1) tetrahydrobioterin (BH4) pathway represents an additional pivotal regulator of ferroptosis, effectively preventing the accumulation of lipid peroxides.^118^ BH4, a pteridine derivative, serves as an endogenous cofactor for numerous enzymes such as nitric oxide synthase (NOS), tryptophan hydroxylase and phenylalanine carboxylase.^119^, ^120^ Biosynthesis of BH4 occurs via a de novo pathway at both peripheral and central levels, commencing with guanosine triphosphate (GTP) and involving three successive enzyme systems: GCH1, 6‐pyruvoyltetrahydrobiopterin synthase (PTPS) and sepiapterin reductase (SR), GCH1 acts as the rate‐limiting enzyme in BH4 synthesis.^121^, ^122^ Furthermore, BH4 functions as a radical‐trapping antioxidant independently from its role as a cofactor,^123^ thus effectively safeguarding lipids against peroxidation.^118^, ^121^, ^122^ Moreover, BH4 can be oxidized to dihydrobiopterin (BH2), and then regenerated by dihydrofolate reductase (DHFR), which plays an essential role in maintaining the BH4/BH2 ratio^124^ (Figure 3).

## 4 FERROPTOSIS IN ISCHEMIC STROKE

**FIGURE 4:**
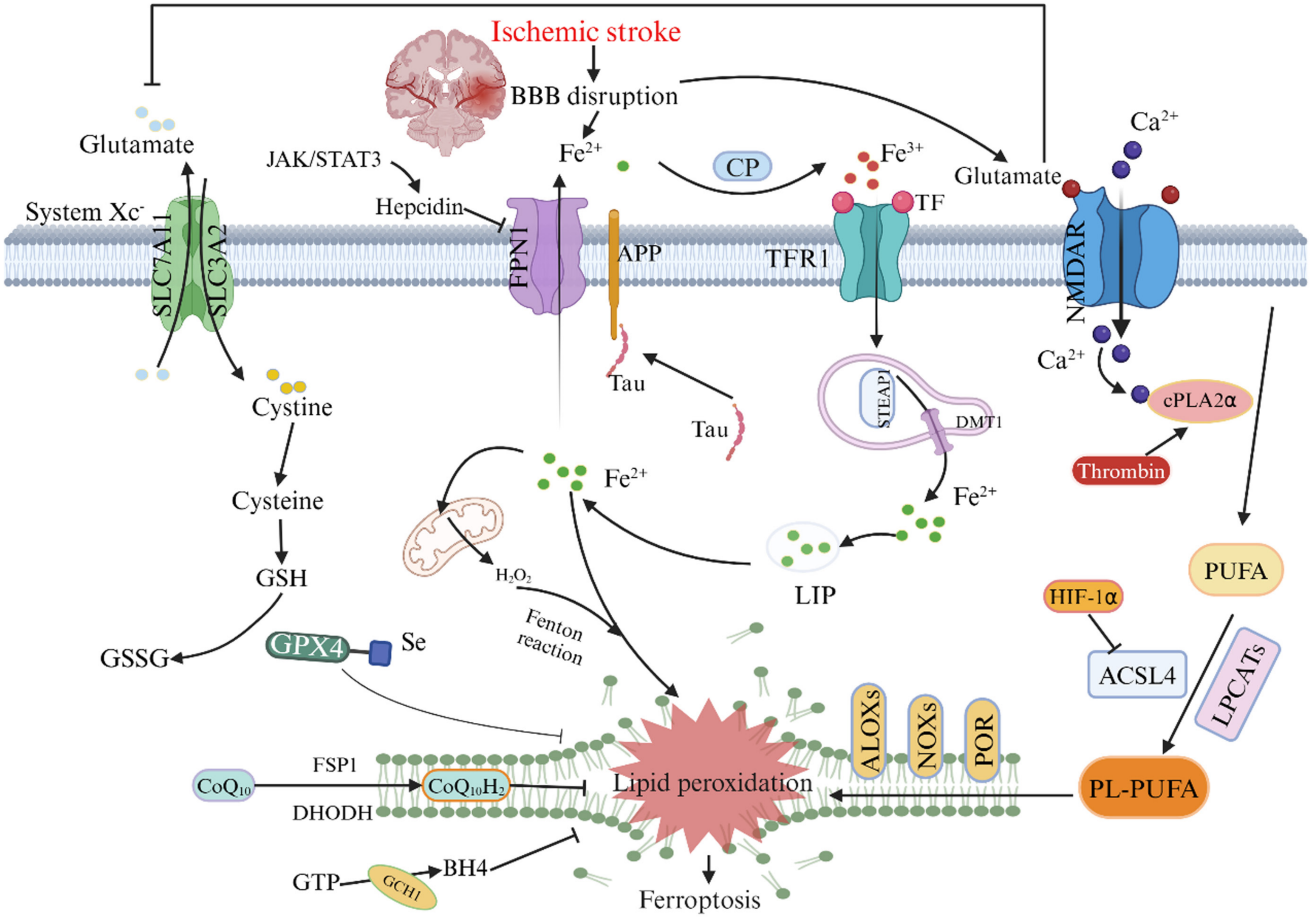
Mechanisms of ferroptosis in ischemic stroke. Accumulation of extracellular glutamate activates NMDAR, facilitates Ca^2+^ uptake, triggers cPLA2α to generate PUFA, and ACSL4 and LPCAT3 convert PUFA to PL‐PUFA. Dysregulation of glutamatergic neurotransmission leads to elevated extracellular glutamate levels, which impede cystine uptake and restrict GSH biosynthesis. Tau‐mediated reduction in APP trafficking and Fpn1 activity hinders iron export. This results in the accumulation of iron in the form of LIP, leading to toxic intracellular iron buildup and ultimately causing neuronal ferroptotic damage following ischemic stroke.

The primary pathological mechanism of IS involves the obstruction of cerebral blood flow. Current clinical treatment strategies focus on expediting the restoration of blood flow as early as possible to save the ischemic penumbra. With prolonged ischemia, there is a gradual exacerbation of neuronal cell injury and clinical symptoms, while brain injury persists during reperfusion.^125^ Inflammation and excitotoxicity can further exacerbate ischemic injury.^126^, ^127^ Research indicated that ferroptosis plays a role in the progression of cerebral ischemia.^128^, ^129^ Recent studies have illustrated that ferroptosis primarily occurs during the reperfusion phase rather than the ischemia phase, as evidenced by a gradual increase in ACSL4 levels, iron content, and malondialdehyde levels with extended reperfusion time, alongside a decrease in GPX4 levels. Notably, these indicators of ferroptosis show no significant changes during ischemia. Furthermore, it has been observed that an iron chelator (deferoxamine) exerts protective effects specifically in tissue subjected to ischemia/reperfusion^130^ (Figure 4).

### 4.1 Iron metabolism dysregulation in ischemic stroke

Serum iron, ferritin, and transferrin saturation levels are correlated with an elevated risk of IS and a poorer prognosis.^131^ Following cerebral ischemia/reperfusion injury (CI/RI), there is dysregulation of iron metabolism leading to iron accumulation in the brain.^132^ The expression of transferrin, ferritin and transferrin receptor in the brain is upregulated under condition of cerebral ischemia and hypoxia.^133^, ^134^, ^135^ Bivalent metal transporter 1 (DMT1) in microglia plays a pivotal role in transferrin‐dependent and non‐transferrin‐dependent iron uptake.^136^ The acidic environment during cerebral ischemia and hypoxia can induce the expression of DMT1 while inhibiting the binding of iron to transferrin, resulting in dissociation between iron and transferrin, thereby increasing brain iron content.^137^, ^138^ Neurons readily uptake this unbound iron, leading to elevated intracellular iron.^139^ Davalos et al.^140^ conducted a study involving 67 cases of acute ischemic stroke which revealed that increased serum ferritin within 24 h of admission was associated with a poor prognosis for stroke patients. Subsequently measuring plasma ferritin at admission in 100 patients with cerebral infarction less than 24 h after onset indicated a significant association between plasma ferritin levels and early neurological impairment as well as stroke progression.^141^

Tau is a protein predominantly expressed in the adult brain, and it plays a crucial role in neurodegenerative diseases such as Parkinson's disease and Alzheimer's disease, as well as stroke.^142^ Several studies have revealed the association between Tau protein and ferroptosis. In 2017, Tuo et al.^6^ illustrated that tau can modulate the trafficking of Amyloid precursor protein (APP), which stabilizes Fpn,^143^ thereby promoting iron exportation and preventing ferroptotic damage.

Ischemia upregulates hepcidin expression through the JAK/STAT3 pathway, leading to FPN degradation and subsequent iron accumulation.^144^ The use of iron chelators like deferoxamine can sequester labile iron pools, reducing brain iron content after ischemic stroke and contributing to decreased neuronal death.^145^, ^146^

### 4.2 Lipid metabolism in ischemic stroke

In addition to adipose tissue, the human brain exhibits the highest lipid index, constituting approximately 60% of the total dry mass of the brain.^147^ Consequently, ischemic stroke can readily induce lipid peroxidation in the brain.^148^ It is well‐established that ischemic stroke generates substantial amounts of ROS, leading to oxidative stress.^149^ Various medicines or biomaterials acting as ROS scavengers have been shown to effectively restore the neurobiological and behavioral functions.^150^, ^151^ Cytoplasmic phospholipase A2α (cPLA2α) is a Ca^2+^ dependent cytoplasmic enzyme that plays a pivotal role in initiation of arachidonic acid (AA) metabolism. Overactivation of NMDA receptors during ischemia can activate cPLA2α by causing excessive Ca^2+^ intake. Thrombin activates cPLA2α to facilitate free AA release.^152^, ^153^

Increasing evidence has demonstrated the involvement of ACSL4 and LOX in ischemic stroke.^154^, ^155^ ACSL4 is a crucial isoenzyme in the metabolism of polyunsaturated fatty acids (PUFAs) such as AA, and it plays a pivotal role in determining the sensitivity to ferroptosis.^156^ Studies have illustrated that oxygen and glucose deprivation (OGD) induces the expression of HIF‐1α, which then binds to the ACSL4 promoter region to inhibit its transcription. Knockdown of ACSL4 can attenuate neuronal cell death, while overexpression of ACSL4 can reduce cell viability, increase lipid peroxidation, and aggravate ischemic brain injury. Ferroptosis inhibitor liproxstatin‐1 can alleviate the adverse reactions caused by overexpression of ASCL4.^128^ Tuo et al.^157^ found that knockout of ACSL4 did not affect the blood flow in the cortex of rats after MCAO/R but reduced infarct volume and alleviated nerve injury by inhibiting ferroptosis. Chen et al.^158^ demonstrated that inhibition of ACSL4 can mitigate the lipid peroxidation associated with ferroptosis after stroke, improve nerve function, and reduce infarct volume. Liu et al.^159^ revealed that calycosin, a phytoestrogen extracted from the root of Astragali radix, connects stably with ACSL4 to suppress lipid peroxidation associated with ferroptosis in ischemic stroke.

LOX catalyzes lipid peroxidation and triggers ferroptosis in numerous diseases.^160^ The expression of 12/15‐LOX is upregulated in neurons around the infarction area of brain, the inhibition of 12/15‐LOX, or the knockout of 12/15‐LOX gene can reduce the infarct size.^155^, ^161^, ^162^ For example, baicalein has been reported to selectively inhibit 12/15‐LOX, thereby offering protection against neuronal cell death following ischemic stroke.^161^

Nrf2 can regulate a wide range of downstream factors, playing diverse roles such as anti‐ferroptosis, anti‐apoptosis, reduction of calcium overload, anti‐oxidative stress, and anti‐inflammatory. It assists in maintaining the REDOX reaction of brain tissue and cells.^163^ Nrf2 plays a crucial role in the management of excessive oxidative stress following ischemic stroke. During ischemia–reperfusion injury, Nrf2 translocates to the nucleus and subsequently activates HO‐1, further inhibiting inflammation.^164^ Apelin, a peptide hormone released from adipose tissue, modulates oxidative stress in many tissues. Duan et al.^165^ illustrated that apelin treatment significantly decreased ROS, lipid peroxidation and inflammation through the AMPK/GSK‐3β/Nrf2 signaling pathway, therefore mediating neuroprotective effects after I/R in the brain. Furthermore, another study showed that Rehmannioside A reduced oxidative stress, diminished infarct volume, and improved cognitive impairment in I/R rats by attenuating ferroptosis through the PI3K/Nrf2/SLC7A11 pathway.^166^

### 4.3 Amino acid metabolism in ischemic stroke

Glutamate serves as the primary excitatory neurotransmitter in the central nervous system (CNS), regulating the neural function and closely related to cell activities, synaptic activity, plasticity, pain perception, learning and memory.^167^, ^168^ The toxic impact of excessive or prolonged extracellular glutamate activation of the excitatory amino acid receptors (particularly NMDARs and AMPARs) is referred to as excitotoxicity.^169^ Glutamate excitotoxicity is widely regarded as one of the mechanisms underlying ferroptosis.^170^ In ischemic stroke, membrane depolarization and potential disruption occur following energy failure, leading to the diffusion of glutamate into the extra‐synaptic region.^171^ Impeded reuptake results in a rapid increase in glutamate levels within ischemic brain areas.^172^ I Inhibiting glutamate release, accelerating its clearance, and blocking its receptors may offer therapeutic benefits for ischemic stroke. For instance, methionine sulfoximine can inhibit the synthesis of glutamine.^173^ mGluR2 and mGluR3 belong to the Group II mGluR family, and their agonists suppress the release of glutamate from presynaptic nerve terminals.^174^ Study have demonstrated that both knockdown of High mobility group box 1 (HMGB1) and suppression of the HMGB1‐TLR4 signaling pathway can increase the expression of glutamate aspartate transporter (GLAST), a specific glutamate transporter, thus enhancing the clearance of glutamate.^175^ *N*‐methyl‐d‐aspartate receptors (NMDARs) are transmembrane proteins, NMDAR channel blockers constitute a class of antagonists with broad clinical and pharmacological significance. Channel blockers usually enter their binding sites only when the channel is open, hence they are also referred to as open channel blockers. NMDAR channel blockers used for treatment include Mg^2+^, ketamine, MK‐801, dextrorphan, memantine, and phencyclidine (PCP).^176^, ^177^, ^178^, ^179^, ^180^ For instance, Ketamine and its metabolites can enhance AMPAR signaling by inhibiting neuronal extrasynaptic NMDARs. AMPARs can stimulate Brain derived neurotrophic factor (BDNF) secretion, thus influencing cell growth and differentiation, cell survival, and synaptic protein transcription.^181^ Study has shown that Glutamate induces acid sphingomyelinase activation and sphingosine accumulation leading to increased generation of ROS which results in the opening of mitochondrial permeability transition pore, the dissipation of mitochondrial transmembrane potential and the rupture of outer membrane, ultimately resulting in system Xc^−^ dependent ferroptosis.^182^ However, studies have found that system Xc^−^ activity rapidly increased after transient middle cerebral artery occlusion (tMCAO) in the rat brain,^183^ and xCT protein levels in microglia/macrophages, neurons, and astrocytes were elevated.^184^, ^185^ Hewett et al.^186^ compared infarcts in mice naturally null for SLC7a11 (SLC7a11^sut/sut^ mice) with wild type (SLC7a11^+/+^) after middle cerebral artery photothrombotic ischemic stroke (PTI) and permanent middle cerebral artery occlusion (pMCAO). They found that the infarct volume in SLC7a11^sut/sut^ mice decreased significantly 48 h after PTI, but no difference between the two groups when MCA was permanently occluded, suggesting that system Xc^−^ aggravated the damage in mice with non‐severe ischemic stroke. In conclusion, IS increased the expression of system Xc^−^ and promotes the export of glutamate from neurons, the excitotoxic effect caused by extracellular glutamate outweigh the protection effect of system Xc^−^.^187^

In addition, glutathione acts as an endogenous ferroptosis inhibitor.^188^ Glutathione levels are depleted in oxidative stress disorders, including stroke, and diminished brain glutathione levels are associated with increased stroke risk. Conversely, replenishing glutathione levels has been shown to reduce neuronal cell death and improve animal function recovery following a stroke. Therefore, maintaining stable intracellular glutathione levels is crucial for ischemic stroke.^189^ GPX4 protein expression was significantly reduced in both vivo and in vitro ischemic stroke models.^190^ Li and colleagues^154^ demonstrated that Baicalin, the main component of *Scuetellaria baicalensis* Georgi root, prevented ferroptotic injury in tMACO mice or OGD/R cells by enhancing the expression of GPX4. Liu et al.^191^ found that edaravone, a free radical scavenger, can reduce infarct volume and functional impairment through anti‐ferroptotic mechanisms such as increasing GSH level and the expression of GPX4.

### 4.4 New hope for IS treatment—inhibition of ferroptosis

Treatment against ischemic stroke by targeting ferroptosis has gained much attention.^192^ Vitexin,^193^ Angong Niuhuang Wan,^194^ selenium,^195^ Srs11‐92,^196^ Se‐methyl L‐selenocysteine,^197^ Rehmannioside A,^166^ Icariside II,^198^ Galangin,^199^ Carvacrol,^200^ Carthamin yellow,^201^ β‐Caryophyllene,^202^ Astragaloside IV,^203^ Salvia miltiorrhiza,^204^ Naotaifang extract,^133^ Naotaifang,^205^ Baicalein,^154^ Caffeic acid,^206^ Danlou tablet,^207^ Rhein,^208^ HSYA and AHSYB,^209^ Calycosin,^159^ Ferrostatin‐1,^210^ Compound Tongluo Decoction,^211^ Loureirin C,^212^ Edaravone,^191^ Quercetin,^213^ Cottonseed oil,^214^ DHT,^215^ Dl‐3‐n‐butylphthalide,^216^ Dimethyl fumarate,^217^ 2‐(1‐(4‐(4‐methylpiperazin‐1‐yl)phenyl)ethyl)‐10H‐phenothiazine,^218^ Kaempferol,^219^ Danhong injection,^220^ Neutral polysaccharide from *Gastrodia elata*,^221^ Resveratrol^190^ alleviate injury after IS by inhibiting ferroptosis (Table 1).

**TABLE 1:** Summary of compounds Interfering with ferroptosis in ischemic stroke.

Authors	Compounds	Study models	Changes	Effects	Mode of action
Guo et al.^193^	Vitexin	OGD/R neuron cells	Inhibit	↓Cell apoptosis; ↓mitochondria injury; ↓SLC7A11; ↑SOD, GSH, Nrf2, Keap1, Tfr1	Keap1/Nrf2/HO‐1
Guo et al.^193^	Vitexin	MCAO/R rats	Inhibit	↑Cell viability; ↓cell apoptosis; ↓neurological Scores; ↓infracted volume; ↓ROS, MDA; ↑SOD, GSH, Nrf2, Keap1, Tfr1; ↓SLC7A11	Keap1/Nrf2/HO‐1
Bai et al.^194^	Angong Niuhuang Wan	MCAO/R rats	Inhibit	↑Nerve function; ↓cerebral infarct volume; ↓mitochondrial dysfunction and morphology disruption, ↑nerve function; ↓cerebral infarct volume; ↑structural integrity of the BBB; ↓ROS, LPO, Fe^2+^; ↑expressions of PPARγ, p‐AKT/AKT and GPX4	↑PPARγ/AKT/GPX4
Shi et al.^195^	Selenium	MCAO/R mice	Inhibit	↓MDA; ↑SOD; ↑GPx4; ↓iron content; ↓p22phox; ↑survival rate of mice; ↓cerebral infarction area; ↓brain cell apoptosis	↓LPO; ↓Fe^2+^
Shi et al.^195^	Selenium	OGD/R N2a cells	Inhibit	↑Cell viability; ↓iron content; ↑GSH; ↑GSH/GSSG; ↑Fth1; ↓Cox2, MDA; ↑SOD, GPx4; ↓p22phox; ↑MMP, Mfn1, mtDNA level	↑Mfn1
Chen et al.^196^	Srs11‐92	MCAO/R mice	Inhibit	↓Neurological deficit score; ↑GSH/GSSG; ↑SOD; ↓ROS. MDA; ↑GPx4, Nrf2, HO‐1	↑Nrf2/HO‐1
Fei et al.^197^	Se‐methyl L‐selenocysteine	MCAO/R mice	Inhibit	↓Infarct volume; ↓brain edema; ↓neurological dysfunction; ↑morphology of neurons; ↑tight junction protein; ↑BBB function; ↑GPX4; ↓ACSL4; ↓4‐HNE; ↑p‐Akt and p‐GSK3β	↑Akt/GSK3β
Fei et al.^197^	Se‐methyl L‐selenocysteine	OGD/R bEnd.3 cells	Inhibit	↑Cell viability; ↑GPX4; ↑BBB function; ↓ACSL4; ↓4‐HNE; ↑Nrf2; ↑p‐Akt and p‐GSK3β	↑Akt/GSK3β
Fu et al.^166^	Rehmannioside A	MCAO/R rats	Inhibit	↓Infarct volume, neurological defects, cognitive impairment; ↑SOD; ↓MDA, MPO, p‐PI3K, p‐Akt; ↑Nrf2, HO‐1, SLC7A11, GPX4	PI3K/Nrf2/SLC7A11
Gao et al.^198^	Icariside II	MCAO/R mice	Inhibit	↓Neurological deficits; ↑sensory motor function; ↓infarct volume; ↑Nrf2 level within nuclei; ↓Nrf2 level within cytoplasm;↑SIRT5, HO‐1, NQO‐1, GPX4, IκBα; ↓the phosphorylation level and activity of NF‐κBp65; ↓mitochondrial dysfunction; ↓mitochondrial ROS, MDA and iron content; ↑NADPH/NADP^+^ ratio, RCI, ATP, GPX4 level, SOD2 activity, SIRT5 activity; ↓IL‐1β, IL‐6, TNF‐α; ↓the number of GFAP‐positive cells	↑Nrf2; ↓NF‐Κb; ↑GPX4
Gao et al.^198^	Icariside II	OGD primary astrocytes	Inhibit	↑Cell viability; ↓LDH; ↓mitochondrial ROS and iron content; ↑RCI, SIRT5, IκBα; ↓IL‐1β, IL‐6, TNF‐α, NF‐κBp65	↑Nrf2; ↓NF‐Κb; ↑GPX4
Guan et al.^199^	Galangin	BCAL/R gerbils	Inhibit	↓Learning and memory deficits; ↑the neurons of CA1; ↓cell death of hippocampal neurons; ↓the hippocampus impairment; ↑GSH, GSHPX, SOD; ↓Fe^2+^, 4‐HNE; ↑SLC7A11, GPX4, H2AX	↑SLC7A11/GPX4
Guan et al.^199^	Galangin	OGD/R hippocampal neurons	Inhibit	↑Survival rate of neurons; ↑SLC7A11; ↓Fe^2+^, ↑GPX4; ↓MDA, H2AX	↑SLC7A11/GPX4
Guan et al.^200^	Carvacrol	BCAL/R gerbils	Inhibit	↑SOD, GSH, GPx4 and Fpn1;↓MDA, TFR1; ↑memory and learning ability; ↓hippocampus impairment	↑GPx4 axis
Guan et al.^200^	Carvacrol	OGD/R Hippocampal neurons	Inhibit	↑GPx4; ↑cell viability; ↓MDA, Fe^2+^	↑GPx4 axis
Guo et al.^201^	Carthamin yellow	MCAO/R rats	Inhibit	↓Neurological deficits, infarction volume, brain water content; ↑MAP‐2; ↑GSH, SOD; ↓MDA, ACSL4 and TFR1; ↑FTH1, GPX4	↑GPx4 axis
Hu et al.^202^	β‐Caryophyllene	MCAO/R rats	Inhibit	↓Neurological deficits; ↓cerebral infarction volume; ↓cerebral pathological damage; ↑NRF2, HO‐1, GPX4; ↓ACSL4, COX‐2; ↑GSH; ↓4‐HNE, MDA	↑NRF2/HO1
Hu et al.^202^	β‐Caryophyllene	OGD/R astrocytes	Inhibit	↑Cell viability; ↑NRF2/HO1; ↓COX2; ↑GPX4	↑NRF2/HO1
Jin et al.^203^	Astragaloside IV	MCAO/R mice	Inhibit	↓infarct size and neurological impairment; ↓MDA, Fe^2+^, Acsl4; ↑GSH	↓Acsl4
Jin et al.^203^	Astragaloside IV	OGD/R neuron cells	Inhibit	↑Cell viability; ↓MDA, LDH, Acsl4 and Fe^2+^; ↑GSH, Slc7a11 and Gpx4; ↑Atf3, Fto	↑Atf3/Fto, ↓Acsl4
Ko et al.^204^	Salvia miltiorrhiza	Transient MCAO mice	Inhibit	↓cerebral infarction volume; ↓brain edema; ↓neuronal loss; ↓neurological deficits; ↓cognitive impairment;↑survival rate of mice;↓iNOS ROS and 4‐HNE; ↑GSH, GPX4, FPN1; ↓ACSL4, Ferritin, iron deposition in the brain	↓LPO; ↑GPX4
Lan et al.^133^	Naotaifang extract	MCAO rats	Inhibit	↓Neurobehavioral scores; ↓iron deposition; ↑GSH; ↓ROS, MDA, TFR1 and DMT1; ↑SLC7A11 and GPX4	↑SLC7A11, GPX4
Liao et al.^205^	Naotaifang	OGD/R BV2 cells	Attenuate	↑Cell viability; ↓Fe^2+^, LPO, ROS, MDA; ↑FPN and GPX4; ↓BMP6, p‐SMADs, p‐STAT3, hepcidin	↓BMP6/SMADs; ↓STAT3
Li et al.^154^	Baicalein	Transient MCAO mice	Inhibit	↓Infarct volume; ↓neurological deficits; ↓Fe^2+^, MDA; ↑GSH, GPX4, ACSL3 and xCT; ↓ACSL4; ↑FTH and mitochondrial ferritin (FTMT)	↑GPX4, ACSL3; ↓ACSL4
Li et al.^154^	Baicalein	OGD/R HT22 cells	Inhibit	↑Cell viability; ↓LDH, ROS and superoxide anion	↓LPO
Li et al.^154^	Baicalein	RSL3 exposed HT22 cells	Inhibit	↑Cell viability; smaller volume, higher mitochondrial membrane electron density and disrupted mitochondrial cristae were ameliorated; ↓ROS, MDA; ↑GSH, MMP, FTH, FTMT, GPX4 and xCT; ↓ACSL4; ↑ACSL3	↑GPX4, ACSL3; ↓ACSL4
Li et al.^206^	Caffeic acid	Permanent MCAO rats	Alleviate	↑Cerebral infarction volume; ↑nerve function; ↑GPx GSH, and SOD; ↑Nrf2, HO‐1, AKT and GSK3β; ↑SLC3A2; ↓TFR1, ACSL4; ↓COX2	↑GPx4; ↑Nrf2
Li et al.^206^	Caffeic acid	OGD/R SK‐N‐SH cells	Alleviate	↓MDA and ROS; ↑GPx4; ↑Nrf2, HO‐1, SLC3A2; ↓ACSL4, TFR1	↑GPx4; ↑Nrf2
Liu et al.^207^	Danlou tablet	Transient MCAO mice	Attenuate	↑Neurological deficits; ↓infarct volume; ↓brain water content; ↓BBB disruption; ↓MDA, GSSG, ROS;↑GSH;↓COX2; ↑GPX4 and SLC7A11	↓LPO; ↑GPX4
Liu et al.^207^	Danlou tablet	OGD/R hy926 cells	Inhibit	↓Cell death; ↓barrier damage; ↑mitochondrial membrane potential; ↓MDA, GSSG, ROS;↑GSH;↓COX2; ↑GPX4 and SLC7A11	↓LPO; ↑GPX4
Liu et al.^208^	Rhein	MCAO/R rats	Inhibit	↓Infarct volume; ↓neurological deficits; ↓BBB disruption; ↑GSH; ↓MDA, Fe^2+^, ROS; ↑GPX4, NRF2, SLC7A11	↑NRF2/SLC7A11/GPX4
Liu et al.^208^	Rhein	OGD/R HT22 cells	Inhibit	↑Cell viability; ↓ROS, MDA, Fe^2+^; ↑NRF2, GPX4, SLC7A11	↑NRF2/SLC7A11/GPX4
Chen et al.^209^	HSYA and AHSYB	OGD/R PC12 cells	Inhibit	↑Cell viability; ↓ROS, Fe^2+^, 4‐HNE, MDA; ↑GSH/GSSG level, GPX4, SLC7A11; ↓mitochondrial morphological damage	↑SLC7A11, GPX4; ↓LPO
Liu et al.^159^	Calycosin	MCAO/R rats	Ameliorate	↓Neurological defect; ↓brain edema; ↓BBB damage; ↓cerebral infarction; ↓Fe^2+^, MDA, ROS; ↑SOD; ↓ACSL4 and TfR1; ↑FTH1 and GPX4	↓ACSL4
Liu et al.^159^	Calycosin	OGD/R PC12 cells	Inhibit	↑Cell viability; ↓cell apoptosis; ↓Fe^2+^, MDA, ROS; ↑SOD; ↓ACSL4 and TfR1; ↑FTH1 and GPX4	↓ACSL4
Liu et al.^210^	Ferrostatin‐1	MCAO/R mice	Inhibit	↓Total iron level; ↑GSH, SLC7A11, GPX4; ↓mitochondrial membrane density; ↑mitochondrial cristae; ↓infarct volume, neurological deficits; ↑p‐AKT and p‐GSK3β	↑AKT
Liu et al.^210^	Ferrostatin‐1	OGD/R HT22 cells	Inhibit	↑Cell viability; ↑SLC7A11, GPX4, GSH; ↓Fe^2+^; ↑p‐AKT and p‐GSK3β	↑AKT
Hui et al.^211^	Compound Tongluo Decoction	MCAO/R rats	Inhibit	↓Cerebral ischemia area; ↓ROS, MDA; ↑SOD; ↓ACSL4, ALOX5; ↑GPX4; ↑angiogenesis; ↑Sonic Hedgehog pathway	Sonic Hedgehog pathway
Hui et al.^211^	Compound Tongluo Decoction	OGD/R PC12 cells	Inhibit	↑Cell viability; ↑Sonic Hedgehog pathway; ↓ACSL4, ALOX5; ↑GPX4;↓ATF4, PERK and cleaved caspase 3; ↑angiogenesis	Sonic Hedgehog pathway
Liu et al.^212^	Loureirin C	MCAO/R mice	Inhibit	↓Neurological scores, brain edema, infarct volume; ↓Fe^2+^; ↑GSH, GPX4, SOD; ↓MDA; ↓mitochondrial membrane density; ↑mitochondrial cristae; ↑Nrf2, HO‐1	↑Nrf2
Liu et al.^212^	Loureirin C	OGD/R SH‐SY5Y cells	Inhibit	↑Cell viability; ↓TFR1; ↑GSH, GPX4, SLC7A11, SOD; ↓DHE, MDA, Fe^2+^, ROS, LPO, Keap1; ↑nuclear Nrf2, GPX4, HO‐1	↑Nrf2
Liu et al.^191^	Edaravone	MCAO/R rats	Inhibit	↑Sensorimotor function; ↓cerebral infarct volume; ↓Fe^2+^, MDA, LPO; ↑GSH, GPX4, total Nrf2, nuclear Nrf2, FPN; ↓IL‐6, IL‐1β, TNF‐α and MPO	↑Nrf2/FPN
Peng et al.^213^	Quercetin	Permanent MCAO rats	Inhibit	↓Infarct volume, neurological scores; ↓Fe^2+^, MDA; ↑GSH, SOD, GPX4, FTH1; ↓ACSL4; ↑Nrf2, HO‐1, nuclear translocation of Nrf2	↑Nrf2/HO‐1
Sun et al.^214^	Cottonseed oil	MCAO/R rats	Attenuate	↓Infarct volume; ↓neurological deficit; ↓BBB disruption, brain water content; ↓transferrin; ↑GPX4, xCT and FTH1; ↓ACSL4; ↑HO‐1, GSH, ROS; ↓MDA, LPO; ↑the proportion of normal mitochondria	↑GPX4; ↓LPO
Wu et al.^215^	DHT	Permanent MCAO rats	Inhibit	↓Infarct volume, neurological score, cerebral edema; ↑Gpx4, GSH, mitochondrial viability; ↓Fe^2+^	↑Gpx4; ↓Fe^2+^
Wu et al.^215^	DHT	t‐BHP PC12 cells	Inhibit	↑Cell viability; ↓ROS, Fe^2+^; ↑Gpx4, GSH/GSSG; ↑MMP; ↓mitochondrial ROS; ↑Nrf2, HO‐1	↑Nrf2/HO‐1
Xu et al.^216^	Dl‐3‐n‐butylphthalide	MCAO/R rats	Attenuate	↓Neurological deficit score; ↓brain water content; ↓cerebral infarct area; ↑neuronal pathology; ↑number of Nissl bodies; ↓neuronal apoptosis, BBB damage; ↑p‐Akt and p‐PI3K; ↓p‐PDGFRβ; ↓TFRC, ROS, MDA; ↑GSH, SLC7A11, GPX4	↑SLC7A11/GSH/GPX4
Yan et al.^217^	Dimethyl fumarate	Chronic cerebral hypoperfusion rats	Inhibit	↓Cognitive impairment; ↓neuronal damage; ↓neuronal loss in hippocampal CA1 regions; ↓Fe^2+^, MDA; ↑GSH, SOD; ↓PTGS2; ↑FTH1, xCT; ↓IL‐1β, TNF‐α, IL‐6, NF‐κB‐p65; ↑IκBα; ↑HO‐1, NQO1, GPX4, NRF2	↑NRF2
Yang et al.^218^	2‐(1‐(4‐(4‐methylpiperazin‐1‐yl)phenyl)ethyl)‐10H‐phenothiazine	MCAO/R rats	Inhibit	↓Cerebral infarction volume; ↓neurological score; ↑GSH; ↓MDA	↑GSH
Yuan et al.^219^	Kaempferol	OGD/R neuron cell	Inhibit	↑SLC7A11, GPX4, nuclear Nrf2; ↓cytoplasmic Nrf2; ↑GSH/GSSG, NADPH/NADP^+^, SOD; ↓Fe^2+^, LPO, 4‐HNE; ↓morphological changes of mitochondria	↑Nrf2; ↓LPO
Zhan et al.^220^	Danhong injection	Permanent MCAO mice	Inhibit	↓Cerebral infarct volume; ↓neurological scores; ↑numbers of Nissl‐positive neurons; ↓TfR1, TF; ↑Ferritin; ↑SATB1, p‐SATB1, SLC7A11, GPX4, HO‐1	↑SATB1/SLC7A11/HO‐1
Zhan et al.^220^	Danhong injection	OGD HT22 cells	Inhibit	↑Cell viability; ↓MDA; ↑SOD, GSH, SATB1, p‐SATB1, SLC7A11, HO‐1	↑SATB1/SLC7A11/HO‐1
Zhang et al.^221^	Neutral polysaccharide from Gastrodia elata	MCAO/R mice	Inhibit	↓Infarct volume; ↓neurological deficit score; ↓cerebral edema, aquaporin 4; ↑GPX4, GSH; ↓Fe^2+^; ↑nuclear NRF2, HO‐1	↑NRF2/HO‐1
Zhang et al.^221^	Neutral polysaccharide from Gastrodia elata	OGD/R HT22 cells	Inhibit	↑Cell viability; ↓ROS, MDA; ↑SOD; ↓Fe^2+^; ↑GSH, SLC7A11, GPX4; ↑NRF2, HO‐1	↑NRF2/HO‐1
Zhu et al.^190^	Resveratrol	MCAO/R rats	Inhibit	↓Mitochondrial damage; ↓cerebral infarct volume; ↓neuronal degeneration; ↓ACSL4; ↑ferritin and GPX4	↓ACSL4; ↑GPX4
Zhu et al.^190^	Resveratrol	OGD/R primary cortical neurons	Inhibit	↑Neuronal viability; ↓Fe^2+^, MDA; ↑GSH; ↓ACSL4; ↑ferritin and GPX4; ↓mitochondrial damage	↓ACSL4; ↑GPX4; ↓Fe^2+^

Abbreviations: AHSYB, anhydrosafflor yellow B; BBB, blood−brain barrier; BCAL/R, bilateral carotid artery ligation/reperfusion; DHE, Dihydroethidium; DHT, 15, 16‐Dihydrotanshinone I; Fth1, Ferritin Heavy Chain 1; GSH, glutathione; GSSG, oxidized glutathione; HSYA, Hydroxysafflor yellow A; Mfn 1, mitofusin 1; MMP, mitochondrial membrane potential; mtDNA, mitochondrial DNA; p‐Akt, Phosphorylated AKT protein; p‐GSK3β, phosphorylated‐GSK‐3 Beta; p‐PI3K, phosphorylation phosphotylinosital 3 kinase; t‐BHP, tert‐Butyl hydroperoxide; TF, transferrin; TfR1, transferrin receptor 1; TFRC, Transferrin receptor.

## 5 CONCLUSION AND PERSPECTIVES

Ischemic stroke is a prevalent condition and represents a primary contributor to both mortality and disability.^222^ The underlying pathological mechanisms remain to be clarified.^223^ Ferroptosis has been implicated in the secondary brain injury associated with ischemic stroke. In the acute phase of ischemic stroke, disruption of the blood–brain barrier leads to aggregation of iron ions in the brain parenchyma, triggering the conversion of hydrogen peroxide to hydroxyl radicals through Fenton reaction, concomitant with reduced GSH levels and increased lipid peroxidation.^224^ This review provides an overview of recent advancements in understanding the pathogenic pathways and regulatory mechanisms of ferroptosis in ischemic stroke, as well as discusses potential therapeutic applications targeting ferroptosis inhibition for treating ischemic stroke. Nevertheless, our understanding of the role of ferroptosis in ischemic stroke remains limited, leaving several questions unanswered. For instance, further clarification is needed regarding the involvement of ferroptosis in neurons, astrocytes, microglia, and oligodendrocytes during ischemic brain injury. Additionally, while other forms of programmed cell death have distinct markers (e.g., caspase activation for apoptosis and gasdermin D for pyroptosis),^225^ specific markers for ferroptosis are still lacking despite current studies being primarily focused on iron levels, GPX4 expression, lipid hydroperoxides, ROS production, and cell viability. Therefore, it is imperative to identify specific markers for ferroptosis. Furthermore, clinical studies investigating ferroptosis in ischemic stroke are warranted given that most research efforts thus far have predominantly centered around cellular and animal models.

## AUTHOR CONTRIBUTIONS

Zhaohui Chai was responsible for writing the manuscript, and Jian Shen and Jiesheng Zheng were responsible for research design. All the authors have contributed to the completion of this paper.

## CONFLICT OF INTEREST STATEMENT

The authors declare that they have no competing interests.

## Data Availability

Data sharing not applicable to this article as no datasets were generated or analysed during the current study.
